# The acceptability of three vaccine injections given to infants during a single clinic visit in South Africa

**DOI:** 10.1186/s12889-016-3324-2

**Published:** 2016-08-08

**Authors:** Hanani Tabana, Lilian D. Dudley, Stephen Knight, Neil Cameron, Hassan Mahomed, Charlyn Goliath, Rudolf Eggers, Charles S. Wiysonge

**Affiliations:** 1 School of Public Health, Community and Health Sciences, University of the Western Cape, Cape Town, South Africa; 2 Division of Community Health, Faculty of Medicine and Health Sciences, Stellenbosch University, Cape Town, South Africa; 3 Metro District Health Services, Western Cape Government: Health, Cape Town, South Africa; 4 School of Nursing and Public Health, College of Health Sciences, University of KwaZulu-Natal, Durban, South Africa; 5 Centre for Evidence-based Health Care, Faculty of Medicine and Health Sciences, Stellenbosch University, Cape Town, South Africa; 6 Department of Immunizations, Vaccines and Biologicals, World Health Organization, Geneva, Switzerland

**Keywords:** Expanded Programme on Immunisation, Three injections, Acceptability, Acceptance, Immunisation coverage

## Abstract

**Background:**

The Expanded Programme on Immunisation (EPI) has increased the number of antigens and injections administered at one visit. There are concerns that more injections at a single immunisation visit could decrease vaccination coverage. We assessed the acceptability and acceptance of three vaccine injections at a single immunisation visit by caregivers and vaccinators in South Africa.

**Methods:**

A mixed methods exploratory study of caregivers and vaccinators at clinics in two provinces of South Africa was conducted. Quantitative and qualitative data were collected using questionnaires as well as observations of the administration of three-injection vaccination sessions.

**Results:**

The sample comprised 229 caregivers and 98 vaccinators. Caregivers were satisfied with the vaccinators’ care (97 %) and their infants receiving immunisation injections (93 %). However, many caregivers, (86 %) also felt that three or more injections were excessive at one visit. Caregivers had limited knowledge of actual vaccines provided, and reasons for three injections. Although vaccinators recognised the importance of informing caregivers about vaccination, they only did this sometimes. Overall, acceptance of three injections was high, with 97 % of caregivers expressing willingness to bring their infant for three injections again in future visits despite concerns about the pain and discomfort that the infant experienced. Many (55 %) vaccinators expressed concern about giving three injections in one immunisation visit. However, in 122 (95 %) observed three-injection vaccination sessions, the vaccinators administered all required vaccinations for that visit. The remaining seven vaccinations were not completed because of vaccine stock-outs.

**Conclusions:**

We found high acceptance by caregivers and vaccinators of three injections. Caregivers’ poor understanding of reasons for three injections resulted from limited information sharing by vaccinators for caregivers. Acceptability of three injections may be improved through enhanced vaccinator-caregiver communication, and improved management of infants’ pain. Vaccinator training should include evidence-informed ways of communicating with caregivers and reducing injection pain. Strategies to improve acceptance and acceptability of three injections should be rigorously evaluated as part of EPI’s expansion in resource-limited countries.

## Background

Vaccines are among the most successful and cost-effective public health interventions available for preventing infectious diseases and deaths in children [1]. Since the World Health Organization (WHO) launched the Expanded Programme on Immunisation (EPI) worldwide in 1974 with six basic antigens (BCG, poliomyelitis (polio), diphtheria, tetanus, pertussis, and measles), there has been significant expansion in the EPI schedule [2]. Depending on the country, a fully immunised child now needs at least six routine immunisation visits to receive between six and 13 antigens in the first year, and from 13 to 20 injections by two years of age. In South Africa (SA), protection is provided against 16 infectious vaccine preventable diseases including measles, mumps, rubella, varicella, hepatitis B, diphtheria, tetanus, pertussis (DTaP), *Haemophilus influenza*e type b (Hib), polio, influenza (flu), rotavirus, and pneumococcal disease [2, 3]. For polio vaccination, in addition to the oral polio vaccine, the injectable polio vaccine is now required globally, and is given in South Africa as a combination vaccine. Despite the availability of combination vaccines, multiple injections are required at several immunisation visits to deliver the recommended antigens. Caregivers (persons who bring children for immunisation) may have concerns about multiple injections at a single immunisation visit [4, 5].

Many low- and middle-income countries (LMICs) are introducing new injectable vaccines, especially in the context of global health initiatives [2]. More infants in LMICs will therefore receive multiple injections during the same immunisation visit, leading to concerns about the acceptability and effect of this practice on EPI outcomes in those countries. While some studies describe the acceptability of multiple vaccine injections in high-income countries [5–7] there is little empiric evidence from LMICs to inform decision making.

In 2009, the South African EPI schedule was revised, with the introduction of among others, pneumoccocal conjugate vaccine (PCV), an injectable given at 6 and 14 weeks. This addition therefore increased the number of injections to three at the 6 and 14 week immunisation visits. The South African Vaccinators Manual also specified that both the PCV and Hepatitis B injections should be administered intramuscularly in the right thigh, and the DTaP-IPV/Hib injection in the left thigh of infants under one year of age [8]_._ The aim of this study was to determine the acceptability and acceptance of three injection vaccinations during a single immunisation visit in SA, to contribute to policy on multiple vaccine injections in LMIC settings.

## Methods

A cross-sectional survey of caregivers of infants and vaccinators at public and private primary healthcare facilities offering EPI services in rural and urban areas in the Western Cape (WC) and KwaZulu-Natal (KZN) provinces of SA was conducted between July and November 2014. Facilities were purposively selected based on service volumes, geographical location and populations served. Prior to selecting facilities, a series of consultations with Municipal and Provincial Departments of Health, and private health service providers were conducted. We sought to include a mix of rural and urban, public and private facilities in the two provinces to achieve a sample representative of the different areas and types of services. We included public clinics that provided a minimum of 200 immunisations per month, and private clinics that provided a minimum of 50 per month. A convenience sample of caregivers 18 years and older with infants aged between six weeks and six months, attending the health services and all health service staff at the selected health facilities who had administered vaccinations within the past year, were invited to participate. The infant age range of 6 weeks to 6 months was chosen to ensure that the sample included infants who were eligible for the three injections at the 6 week and 14 week immunisation visit, and that the time period since the last three injection immunisation was not longer than 3 months. A sample size estimate of 200 caregivers was based on the 2012/2013 national immunisation coverage of 80 and 5 % precision [9]. A sample of 50 vaccinators from each province was estimated based on the number of staff providing immunisations at the selected facilities. A pilot study was conducted to improve the validity of the data collection tools and procedures. Both quantitative and qualitative data was collected using questionnaires which included closed and open-ended questions for caregivers were translated into the key languages of the two provinces (English, Afrikaans, *isiXhosa*, and *isiZulu*) and administered by trained fieldworkers in the language of the caregiver. The vaccinators are fluent in English and were interviewed in English by trained fieldworkers. Both caregiver and vaccinator interviews were interviewer administered. Caregivers were interviewed after the infant had received the vaccination or other services at the clinic while vaccinators were interviewed at the facility at a time convenient to them. An observation checklist was used to record actual practices of vaccinators during the administration of three vaccine injections. Informed consent was obtained from all participating caregivers and vaccinators. None of the caregivers or vaccinators invited to participate in the study refused to.

Acceptability and acceptance of three vaccine injections at one immunisation visit were the main outcomes assessed. Acceptability refers to the adaptation of care to the wishes, expectations and values of caregivers. Acceptability was measured as the caregivers’ and vaccinators’ knowledge, perceptions of benefits and expressed preferences regarding three vaccine injections during a single immunisation visit. Acceptance has been defined as ‘compliance with vaccinations by a public which yields to the recommendations and social pressure of health workers and community leaders’[10]. Acceptance was assessed in caregivers by the expressed willingness to allow their infant to receive three injections during a single visit; and the extent to which the infant actually received the injections at one visit. Assessed in vaccinators by expressed willingness to provide three injections in one visit; and the extent to which vaccinators actually provided their injections according to existing EPI norms and standards.

### Data analysis

The questionnaires were loaded using data collection software onto mobile phones. Fieldworkers entered responses to questions directly into the phones using the keypad. The data collected were transferred on a daily basis into a central Microsoft Access database for quality checks and data storage. The cleaned data were exported to STATA version 13 (Statacorp LP, Texas, USA) for further processing and analysis. Two sided Chi-square and t-tests were used to assess associations and differences and *p*-values less than 0.05 were considered statistically significant. Qualitative data from open-ended questions were analysed and responses thematically categorised. Qualitative results are reported both as frequency of responses around content, and with quotes which represent the themes that emerged.

## Results

### Caregiver and vaccinator characteristics

A study sample of 229 caregivers from 15 rural and urban clinics in two provinces of South Africa was used to investigate the acceptability of three vaccine injections (Table 1). Most of the caregivers were the infants’ parent (93 %), female (99 %), single (65 %), and had 8–12 years of formal school education (85 %). Fifty nine percent of the infants were aged 4–6 months, and 51 % were female. Most (71 %) attended the clinic for an immunisation visit, which was the first (6 week) three-injection immunisation visit for 41 % of the infants (Table 1). At the ‘study’ immunisation visit, 138 (60 %) infants received three vaccine injections.

**Table 1:** Characteristics of 229 caregivers and 98 vaccinators (healthcare providers)

Caregiver (*N* = 229)	no.(%)	Vaccinator (*N* = 98)	no.(%)
Age (years)		Age	
<25	92 (40.2)	<40	41(41.8)
≥25	137(59.8)	≥40	57 (58.2)
Gender		Gender	
Male	3 (1.3)	Male	9 (9.2)
Female	226 (98.7)	Female	89 (90.2)
Relationship to child		Position	
Parent	212 (93)	Professional nurse	75 (76.5)
Other	17 (7.0)	Enrolled nurse	21 (21.4)
		Other	2 (2.0)
Education		Experience administering EPI	
Tertiary	26 (11.4)	<1 year	15 (15.3)
Matric/High School	195 (85.1)	1–5 years	36 (36.7)
Primary/None	8 (3.5)	>5 years	47 (50.0)
Marital status		EPI training	
Married	63 (27.5)	Yes	76 (77.6)
Single	149 (65.1)	No	22 (22.4)
^a^Other	17 (7.4)		
Caregiver Infant Age			
6 weeks	93 (40.8)		
4–6 months	135 (59.2)		
Other (age missing)	1 (0.0)		
Gender			
Male	112 (48.9)		
Female	117 (51.1)		
Reasons for visiting the clinic today			
Immunisation visit	163 (71.2)		
Other (non immunisation)	66 (28.8)		
Number of injections received at visit			
1 injection	4 (1.75)		
2 injections	14 (6.11)		
3 injections	138 (60.26)		
Don’t know	1 (0.44)		
Not applicable	72 (31.44)		

^a^Life partner/widowed/divorced

Ninety eight vaccinators participated of whom 77 % were professional nurses and 91 % female, with a median age of 43 years (range 25 to 69 years) (Table 1). The vaccinators were experienced, with 86 % having administered vaccines for a year or longer and 50 % had more than five years of vaccination experience. Most (78 %) vaccinators had received training in the EPI, although only 15 % had been trained in the last year.

Most vaccinators (99 %) felt it was very important to provide information about three- injection vaccinations to caregivers, but only 55 % said they always provided explanations about the reasons for multiple injections (Table 2).

**Table 2:** Acceptability and acceptance of multiple injections given at a single visit to caregivers and vaccinators

Caregivers (*N* = 229)	n (%)	Vaccinator (*N* = 98)	n (%)
Caregivers understanding of immunization		Concern about giving multiple injections	
To protect from disease	133 (58.1)	Concerned	54 (55.1)
To keep babies healthy	69 (30.1)	Not concerned	44 (44.9)
To prevent epidemics	26 (11.4)	Reason for concerns	
Other	1 (0.4)	Side effects	6 (11.1)
		Crying & pain	32 (59.3)
		Difficulty holding child	2 (3.7)
Caregiver informed about number of injections during vaccination session		Caregiver not coming back	10 (18.5)
Yes	162 (70.7)	Don’t know enough about why Immunization given	3 (5.6)
No	67 (29.3)	Parent objection	1 (1.9)
No. of injections perceived ‘too many’		No. of injections perceived ‘too many’	
<3 injections	20 (8.7)	<3 injections	2 (2.0)
≥3 injections	196 (85.6)	≥3 injections	94 (96.0)
Uncertain	13 (5.7)	Uncertain	2 (2.0)
Caregivers knowledge of diseases immunisations prevent		Caregivers of babies (6 weeks old) expressing unhappiness about multiple injections	
Pneumonia (Pnuemococcal or Hib)	3 (1.3)	Always	20 (20.4)
Diarrhoea (rotavirus)	8 (3.5)	Often	36 (36.7)
Measles	28 (12.2)	Sometimes / Seldom	22 (22.5)
Polio	77 (33,6)	Seldom	12 (12.2)
Hepatitis B	4 (1.8)	Never	8 (8.2)
Diptheria	6 (2.6)	Caregivers of babies (older than 6 weeks) expressing unhappiness about multiple injections	
Tetanus	3 (1.3)	Always/often	19 (19.4)
Whooping cough (Pertussis)	11 (4.8)	Sometimes / Seldom	50 (51.0)
TB	89 (38.9)	Never	29 (29.6)
Satisfaction with injections administered at visit			
Satisfied	213 (93.0)		
Dissatisfied	16 (7.0)		
Satisfaction with vaccinators at visit		Importance of providing more information to caregivers about immunisations	
Satisfied	221 (96.5)	Very important	97 (99.0)
Dissatisfied	8 (3.5)	Somewhat important	1 (1.0)
Preferred number of visits		Frequency of explaining the reasons for multiple injections to caregiver	
One visit for 3 injections	166 (72.3)	Always	54 (55.1)
More visits for fewer injections each	59 (25.8)	Often	26 (26.5)
Other	4 (1.8)	Sometimes	16 (16.3)
Ever told to come for more visits for less injections		Seldom	2 (2.1)
Yes	35 (15.3)	Advised caregivers to bring child for extra visits for less injections	
No	193 (84.3)	Always / often	10 (10.2)
Uncertain	1 (0.4)	Sometimes / seldom	9 (9.2)
		Never	79 (80.6)
Caregiver informed about the number of immunization injections infant would receive		Copy of protocol/ guideline seen	
Yes	162 (70.7)	Protocol in immunization room	83 (87.4)
No	67 (29.3)	Protocol in facility / other room	10 (10.5)
Where were injections given		No protocol seen	2 (2.1)
Combination RRL (2 x right thigh & 1 left thigh)	120 (52.4)	Protocol Used	
Combination LLR (2 x left thigh & 1 right thigh)	16 (7.0)	Yes	95 (96.9)
Upper arms	1 (0.4)	No	3 (3.1)
Other	1 (0.4)		
Can’t remember	1 (0.4)		
Not applicable (no injection given)	64 (28.0)		
<=2 injections	26 (11.4)		
Proportion of infants who are up to date for age on immunisations (ie acceptors)			
Yes	220 (96.1)		
No	7 (3.1)		
Patient held immunisation record not available	2 (0.8)		

## Acceptance and acceptability of multiple immunisation injections to caregivers and vaccinators

### Perceived importance of immunisations

Caregivers’ main sources of information about immunisation were nurses or doctors (72 %) and family (7 %). Their understanding of the purpose of immunisation was to protect the infant from disease (88 %), and prevent the spread of infection (11 %) (Table 2). Few caregivers knew the specific vaccine preventable diseases of the EPI (SA) schedule, with only TB (39 %), polio (33,6 %) and measles (12 %) often mentioned by caregivers. Despite this, most (205/229 -90 %) caregivers believed that immunisation was very important to their infant’s general health as expressed in the following quotes;

“*Because I know that it is good for the child’s health and well-being”*

“*If the child gets all the injections while little they won’t get ill when they are bigger”*

### Number of vaccines allowable during a single immunisation visit

Most (71 %) caregivers knew about the number of vaccination injections to be administered. Eighty six percent felt that three or more injections were too many to be given to infants per immunisation visit (Table 2), mainly because of the pain experienced by the infant (52 %).

“*It’s too painful and leads to sleepless nights for the infant”*

“*The infant is too young and it felt like they are in deep pain when they were injected”*

However, if three vaccine injections were required, most caregivers (72 %) preferred one immunisation visit for the three injections (Table 2). Despite feeling that three or more injections were too many per immunisation visit, 97 % of caregivers were willing to bring their infant for three-injection vaccination visits again, or to recommend that others bring their infants for three-injection vaccination visits (99 %).

Reasons given for caregivers’ willingness to bring infants for three-injection vaccination visits in the future were mainly to improve the infant’s health (49 %), and to protect against diseases (38 %).

“*I only do it for the child’s sake because I know that he will be safe from getting sick”*

“*To protect my child from diseases that attack little babies”*

The benefit of immunisations for the infants’ health (53 %) and protection against disease (41 %) were also the main reasons for recommending three injections at one immunisation visit to others. Although caregivers were willing to bring their child for three-injection vaccination visits again, or to recommend three vaccine injections at a single visit to others, they also expressed the need for changes such as reducing the number of injections per immunisation visit by combining injections (31 %) or substituting injections with oral vaccines (20 %).

### Satisfaction with vaccinations

Almost all (97 %) caregivers were satisfied with vaccinator’s EPI services and 93 % expressed satisfaction with the vaccination injections given to their infants (Table 2). Satisfaction with injections did not differ between caregivers of infants attending for the first three-injection vaccination visit (at 6 weeks of age) and those with older infants who had been exposed to two immunisation visits for three injections (*P* = 0.19).

The infant’s response to the injection (12 %) contributed to the level of satisfaction with the immunisation services, with dissatisfied caregivers indicating that the infant’s emotional response and possible side effects were important factors.

Caregivers of smaller (six-week old) infants were more dissatisfied with the vaccinator’s care than caregivers of older infants (*P* <0.05) (Table 3). Vaccinators also reported that caregivers of six-week old infants expressed unhappiness about the three-injection vaccinations ‘always or often’ (57 %) compared to caregivers of babies older than six weeks who expressed such unhappiness less frequently (19 %) (Table 2).

**Table 3:** Associations between caregiver and infant characteristics with selected variables measuring acceptability and acceptance of multiple injections to caregivers and vaccinators

	Cross variable (*n* = 229)						
Variable (*n* = 229)	Satisfaction with injection	Satisfaction with vaccinator	Will recommend multiple injections	Will come for multiple injections again	Injections too many	Caregiver understanding of immunization purpose	Caregiver knowledge of diseases immunised against
Caregiver	Chi2 P-value	Chi2 P-value	Chi2 P-value	Chi2 P-value	Chi2 P-value	Chi2 P-value	Chi2 P-value
Age	0.821	0.564	0.339	0.792	0.086	0.158	0.022
Gender	0.633	0.740	0.987	0.775	0.106	0.567	0.011
Relationship to child	0.422	0.577	0.002	<0.001	0.213	0.676	0.612
Education	0.677	0.859	<0.001	<0.001	0.803	0.879	0.699
Marital status	0.383	0.297	0.256	0.607	0.249		
Reasons for visiting clinic today	0.824	0.300	0.665	0.805	0.058	0.253	0.873
Infant							
Infant age	0.656	0.007	0.343	0.642	0.839	0.713	0.164
Infant gender	0.669	0.434	0.381	0.439	0.674	0.138	0.512
Number of injections received at visit	0.469	0.243	0.995	0.967	0.438	<0.001	0.560
	Cross variable (*n* = 98)						
Vaccinator characteristics (*n* = 98)	Concern about giving 3 injections	Reasons for concerns	Injections too many	Unhappiness from caregivers of infants 6 week old	Unhappiness from caregivers of infants >6 weeks	Information giving importance	Protocol Used
	Chi2 P-value	Chi2 P-value	Chi2 P-value	Chi2 P-value	Chi2 P-value	Chi2 P-value	Chi2 P-value
Age	0.867	0.025	0.469	0.786	0.739	0.236	0.136
Gender	0.500	0.705	0.810	0.285	0.238	0.749	0.576
Experience administering EPI	0.403	0.788	0.340	0.705	0.340	0.578	0.187
Years of EPI training	0.436	0.232	0.877	0.873	0.780	0.864	0.639

Caregiver satisfaction with vaccinators was influenced by the vaccinators’ handling of the infant (36 %) and attitude towards the caregiver (33 %).

“*I am happy with the sister’s [professional nurse] positive attitude but besides generally the staff at this clinic is friendly. I am supposed to use [x] Clinic but I decided to use this clinic because of positive staff attitudes”.*

Caregivers also indicated that communication by vaccinators (15 %) and the competency of vaccinators (9 %) contributed to the level of satisfaction with the vaccinators.

“*She is patient; she does not shout at us and she answers our concerns and questions so well”*

### Compliance with the immunisation guidelines

An important measure of acceptance is the extent to which the infants have completed all immunisations required for their age. We found that 220 (96 %) of the infants were up to date for age for their immunisations based on the patient-held immunisation records (Table 2).

In terms of compliance with EPI policy, 95 % vaccinators were able to produce the standard written protocols for vaccinations (Table 2), and 99 % of injections were given in the infants’ thighs as prescribed by the National EPI [SA] policy [8] (Table 2).

However, 15 % of infants were vaccinated while lying unsupported on the examination couch, contrary to the national policy which recommends that the infant be securely held on an adult’s lap [8]. A few (10 %) vaccinators also regularly advised caregivers to bring their infants for extra immunisation visits to have fewer vaccinations at each immunisation visit (Table 4).

**Table 4:** Vaccinator practices – observed (*N* = 129 observations)

Variable	Categories	n (%)
Greeted and made eye contact with carer	Yes	120 (93.0)
	No	9 (7.0)
Made friendly contact with infant	Yes	112 (86.8)
	No	17 (13.2)
Reassured/ encouraged the caregiver	Yes	89 (69.0)
	No	40 (31.0)
Explained the importance of the infant being fully immunized	Yes	69 (53.5)
	No	60 (46.5)
Explained the procedure clearly	Yes	84 (65.1)
	No	45 (34.9)
Explained what was expected of the caregiver during the procedure	Yes	91 (70.5)
	No	38 (29.5)
Provided answers the caregiver seemed satisfied with	Yes	41 (31.8)
	No	5 (3.9)
	Not applicable	83 (64.3)
Infants position during immunization	Baby lying on bed	19 (14.7)
	Baby on caregivers lap	108 (83.7)
	Another health worker holding the baby	1 (0.8)
	Other	1 (0.8)
Did the caregiver seem upset by the 3^rd^ injection	Yes	27 (20.9)
	No	102 (79.1)
Reassured the caregiver during the procedure	Yes	80 (62.0)
	No	49 (38.0)
Provide counseling about common side effects	Yes	26 (20.2)
	No	103 (79.8)
Informed the caregiver when to return for the next immunization	Yes	66 (51.2)
	No	63 (48.8)
Administered all vaccines due at this visit	Yes	122 (94.6)
	No	7 (5.4)

Researchers observed the administration of 129 three vaccine injections (Table 4). Vaccinators explained the importance of full immunisation of infants to 54 % of caregivers, explained the procedures to 65 % of caregivers, provided counselling on side effects to 20 % of caregivers and informed 51 % of caregivers when to return for the next immunisation visit. In 122 (95 %) of the 129 observed vaccinations (Table 4), the vaccinators administered all the vaccines that were due on that immunisation visit. The seven injections that were not administered were due to stock-outs of particular antigens, and caregivers were advised to return for those. Vaccinator years of experience providing EPI services was not associated (*P* = 0.87) with suggestions for the child to be brought in for extra immunisation visits instead of administering all recommended injections during a single immunisation visit in efforts to reduce pain and discomfort.

### Vaccinator concerns regarding multiple injections

Many (55 %) vaccinators expressed some concern about giving three injections in one immunisation visit, with their greatest concern being the crying and pain (59 %) experienced by infants. Although vaccinator age was significantly associated with the years of vaccination experience (*P* < 0.05), vaccinator age was not a significant factor of acceptability (concerns about three vaccine injections given during a single immunisation visit) (*P* = 0.87), or willingness to give all recommended vaccinations per immunisation visit (*P* = 0.62). Further, vaccinators’ concerns were not associated with: number of injections perceived as too many (*P* = 0.98), or years of experience administering EPI vaccines (*P* = 0.40).

None of the vaccinators was concerned about one or two injections given during a single immunisation visit.

The main challenges vaccinators reported when giving multiple injections included caregivers’ (21 %) and infants’ (15 %) emotional responses and their own concerns about high risk infants (10 %).

“*When the mums are tense, it makes it difficult. Also just seeing the baby cry breaks my heart”*

“*…also if the mother doesn’t want the injection. Also when babies are premature and abnormal babies e.g. physical disabilities.”*

Vaccinators’ suggestions for improving the acceptability of three-injection vaccination visits included giving more caregiver education (59 %) and fewer injections (41 %). For fewer injections, several vaccinators recommended combining injections and changing the mode of vaccine delivery to oral vaccination.

“*Educating moms on a regular basis makes the process better”*

### Other factors associated with caregiver and vaccinator attitudes, perceptions and practices

The number of injections perceived as too many was not associated with whether or not caregivers would come for a next immunisation visit (*P* = 0.59) or their age (*P* = 0.09). Further, caregiver age was not significantly associated with willingness to recommend three injections per immunisation visit to others (*P* = 0.34), willingness to come for three injections in the future (*P* = 0.79) and whether or not caregivers were satisfied with injections (*P* = 0.82) (Table 3).

## Discussion

With the increasing complexity of global childhood immunisation services, caregivers’ perceptions, knowledge and understanding becomes a vital element in the success of the EPI.

This study indicates that almost all caregivers bringing infants for immunisation in SA accepted three vaccination injections at a single visit. Despite high levels of concern about the pain and discomfort experienced by infants receiving three injections, they strongly preferred a single visit to returning to complete the scheduled number of vaccines required.

The high proportion of single mothers in this survey is in line with the SA population. Only 31 % of mothers of children under five are legally married, and 67 % of SA birth registrations do not include information about fathers [11, 12]. Study caregivers had a 10 % higher level of secondary education than mothers of children aged 0–4 in the general population [11]. This is consistent with previous studies from South Africa and sub-Saharan Africa where caregivers with secondary or higher education levels were more likely to have their children immunized compared to those with lower education levels [13, 14].

Approximately half of caregivers and vaccinators felt that three vaccine injections were too many at one immunisation visit. Acceptability is influenced by the clients’ perceptions of the benefits versus the risks or costs of the care provided [15]. Most caregivers had a basic understanding of the purpose of immunisation, but limited knowledge of the vaccine-preventable diseases that their infants are immunised against as part of the EPI [SA] schedule. Many caregivers were not informed about or prepared for the three vaccine injections at one visit. Although vaccinators providing EPI services recognized the importance of giving caregivers appropriate information about immunisation, vaccinators only sometimes provided relevant information. Caregivers indicated that more information was needed for them to understand more about immunisation of their infants.

A systematic review of interventions for improving coverage of childhood vaccinations in LMICs reported that there was moderate-certainty evidence that health education (community based, facility based and facility plus reminders) improves immunisation coverage [16].

While caregivers expressed preference for fewer injections, they were largely satisfied with the three injections and vaccinators’ care. However, to reduce the perceived risks or discomforts of multiple injections, better pain management for the infant during the vaccination could improve the acceptability of multiple injections. High quality evidence-based support interventions such as breastfeeding or administration of a sucrose solution to the infant during vaccination injection, not placing the infant supine but holding the infant comfortably and securely upright during the procedure, and other universal psychological injection pain minimisation techniques could limit the discomfort experienced by infants and their caregivers [17–19]. South Africa was due to introduce hexavalent (DPT-HepB-Hib-IPV) vaccine from June 2015, reducing the number of injections to two again (http://apps.who.int/immunization_monitoring/globalsummary/countries?countrycriteria%5Bcountry%5D%5B%5D=ZAF).

The caregivers in this study demonstrated a high acceptance of three vaccine injections at one immunisation visit and a willingness to return for three injections in the future. Although the acceptability of three injections was a concern for vaccinators, this generally did not affect their practices, with vaccinators demonstrating a high compliance with the policies and the administration of vaccines according to the EPI [SA] schedule [8]. Acceptance of three vaccine injections at one immunisation visit was high, despite several significant differences in the profile of caregivers and vaccinators, their practices and the acceptability of three injections at one immunisation visit.

### Implication of the findings

Poor understanding of the reasons for immunisations could be an important contributor to the high burden of non-immunized children in parts of sub-Saharan Africa [14]. Further increasing the number of vaccination injections may have implications for EPI acceptability and immunisation coverage in many LMICs. Although acceptance of three vaccine injections was high for caregivers attending health services in South Africa, the lower acceptability during the first three-injection vaccination session is a concern. Innovative strategies for educating caregivers on vaccinations are needed, particularly for caregivers with less education or who do not regularly attend health services. The high level of concern about the pain and distress experienced by infants should also be included in EPI guidelines and addressed in the training of vaccinators by including evidence-based practices for reducing pain in the administration of vaccine injections [17, 18]. These measures are particularly important for younger infants coming for the first three-injection vaccination visit.

Further research using appropriate study designs, is needed to assess the factors contributing to the acceptability and acceptance of multiple vaccine injections during a single immunisation visit in different settings. In addition, research on strategies to improve acceptability and acceptance of multiple injections in LMIC’s should be undertaken. New combination vaccines are needed to reduce the number of vaccine injections needed per immunisation visit.

### Limitations

The sampling of facilities was stratified to ensure geographic and socio-economic representation from the two provinces. However, due to recruitment problems the study included more public sector and Western Cape participants. Although a high proportion of infants were up to date for their vaccinations, the study represented caregivers attending health facilities for EPI or other services there may have been a selection bias and may not have detected infants who failed to return for subsequent vaccinations. Thus, the included sample may be biased and not representative of the population of caregivers in South Africa, especially those who do not attend health facilities regularly.

## Conclusions

This study found no evidence of reduced acceptance by caregivers and vaccinators of three injections at a single immunisation visit amongst health service attenders in South Africa. However, the acceptability of three injections was a concern for both caregivers and vaccinators. Acceptability for caregivers was influenced mainly by the infant’s pain and distress. This was exacerbated by limited information, communication and education about vaccines and the number of injections. Acceptability of three injections may be improved through enhanced vaccinator-caregiver communication, and improved management of infants’ pain. Vaccinator training should include evidence-informed ways of communicating with caregivers and reducing injection pain. Strategies to improve acceptance and acceptability of three injections should be rigorously evaluated as part of the expansion of the EPI in low and middle-income countries.
